# Cell transfer technology for tissue engineering

**DOI:** 10.1186/s41232-017-0052-7

**Published:** 2017-10-16

**Authors:** Keiko Akazawa, Kengo Iwasaki, Mizuki Nagata, Naoki Yokoyama, Hirohito Ayame, Kazumasa Yamaki, Yuichi Tanaka, Izumi Honda, Chikako Morioka, Tsuyoshi Kimura, Motohiro Komaki, Akio Kishida, Yuichi Izumi, Ikuo Morita

**Affiliations:** 1 Department of Periodontology, Graduate School of Medical and Dental Sciences, Tokyo Medical and Dental University (TMDU), 1-5-45 Yushima, Bunkyo-ku, Tokyo, 113-8510 Japan; 2 Department of Nanomedicine (DNP), Graduate School of Medical and Dental Sciences, Tokyo Medical and Dental University (TMDU), 1-5-45 Yushima, Bunkyo-ku, Tokyo, 113-8510 Japan; 3 Life Science Laboratory, Research and Development Center, Dai Nippon Printing Co., Ltd., 1-1-1 Kaga-cho, Shinjuku-ku, Tokyo, 162-8001 Japan; 4 Department of Comprehensive Reproductive Medicine, Graduate School of Medical and Dental Science, Tokyo Medical and Dental University, 1-5-45 Yushima, Bunkyo-ku, Tokyo, 113-8510 Japan; 5 Department of Pediatrics and Developmental Biology, Graduate School of Medical and Dental Science, Tokyo Medical and Dental University (TMDU), 1-5-45 Yushima, Bunkyo-ku, Tokyo, 113-8510 Japan; 6 Department of Material-based Medical Engineering, Institute of Biomaterials and Bioengineering, Tokyo Medical and Dental University (TMDU), 2-3-10, Kanda-Surugadai, Chiyoda-ku, Tokyo, 101-0062 Japan; 7 Department of Cellular Physiological Chemistry, Graduate School of Medical and Dental Sciences, Tokyo Medical and Dental University (TMDU), 1-5-45 Yushima, Bunkyo-ku, Tokyo, 113-8510 Japan

**Keywords:** Cell-based therapy, Cell transfer, Cell transplantation, Regeneration

## Abstract

We recently developed novel cell transplantation method “cell transfer technology” utilizing photolithography. Using this method, we can transfer ex vivo expanded cells onto scaffold material in desired patterns, like printing of pictures and letters on a paper. We have investigated the possibility of this novel method for cell-based therapy using several disease models. We first transferred endothelial cells in capillary-like patterns on amnion. The transplantation of the endothelial cell-transferred amnion enhanced the reperfusion in mouse ischemic limb model. The fusion of transplanted capillary with host vessel networks was also observed. The osteoblast- and periodontal ligament stem cell-transferred amnion were next transplanted in bone and periodontal defects models. After healing period, both transplantations improved the regeneration of bone and periodontal tissues, respectively. This method was further applicable to transfer of multiple cell types and the transplantation of osteoblasts and periodontal ligament stem cell-transferred amnion resulted in the improved bone regeneration compared with single cell type transplantation. These data suggested the therapeutic potential of the technology in cell-based therapies for reperfusion of ischemic limb and regeneration of bone and periodontal tissues. Cell transfer technology is applicable to wide range of regenerative medicine in the future.

## Background

Recent progress in tissue engineering made it possible to treat various diseases using ex vivo expanded cells [1]. The possibility of the cell-based therapy for many diseases has been widely studied. The selection of cell culture methods, which facilitate therapeutic effect of the cells, and methods of transplantation, which include the ideal carrier for the local transplantation, are essential considerations in cell-based therapy [2]. We have developed novel cell transplantation method “cell transfer technology,” utilizing photolithography, which is often used for micropatterning formation in semiconductor manufacturing and printing [3]. This technology allows us to transfer cultured cells onto scaffold material, like pictures and letters printed on a paper. We have investigated the possibility of this novel method for cell-based therapy using several disease models. In this review, we outline the cell therapies that we have reported so far using the cell transfer technique.

### Cell transfer using photolithography

Photolithography is a word with a prefix “photo” meaning light to “lithography,” which is originated from lithograph. Literally, among various lithographic methods, photolithography uses the pattern made by light for document copy. Due to its precision, reproducibility, and mass productivity, photolithography is widely used in the precision machinery industry and printing. Photolithography consists mainly of two steps, namely the depiction of desired pattern on the substrate and “transfer” of the pattern to the product surface.

**Fig. 1:**
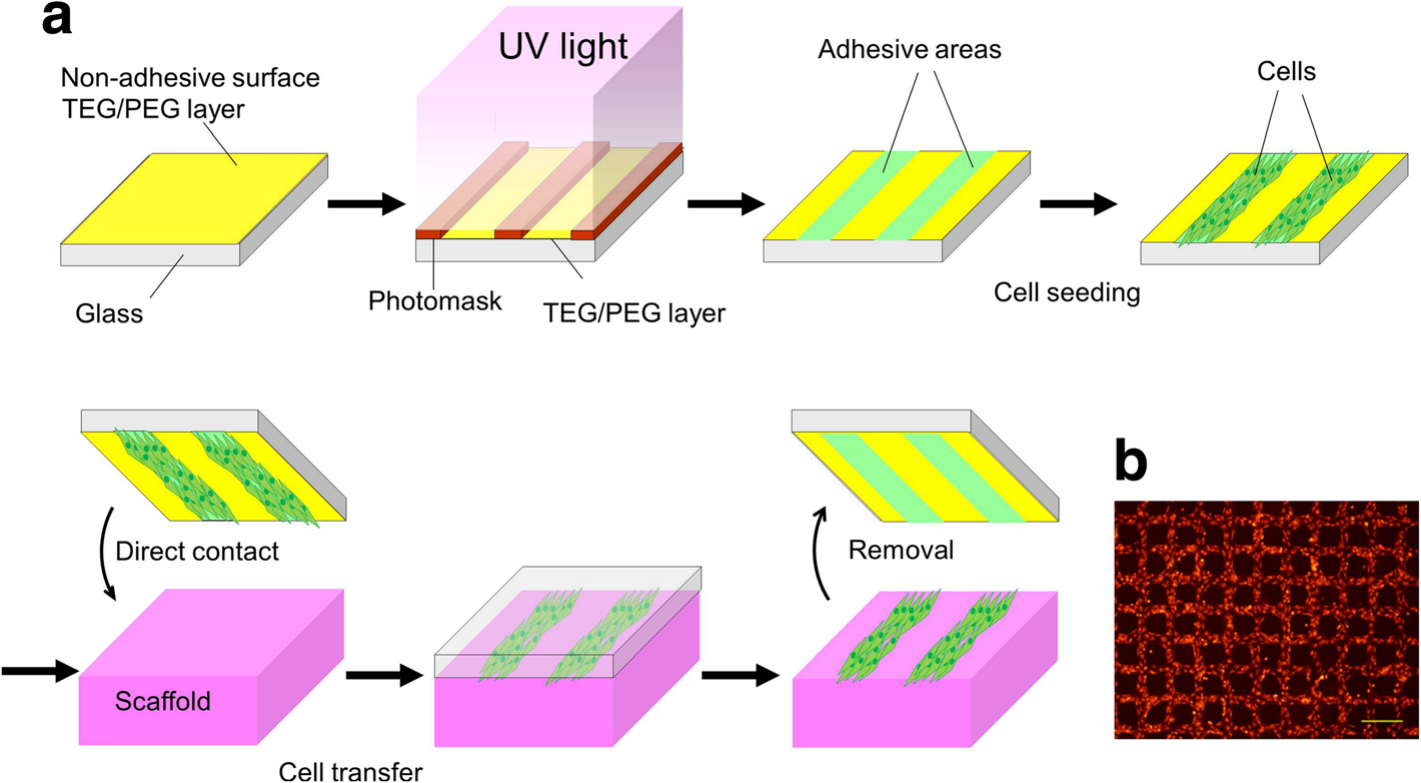
Schema of cell transfer technology. **a** Procedure of cell transfer technology from construction of the transfer substrate to cell transfer. TEG/PEG layer (yellow) is formed on glass substrate. Following pattern drawing (photomask: red), UV light is radiated on the substrate. The surface exposed to UV light becomes cell adhesive area (green). Several hours of incubation after cell seeding on the substrate, the cells are transferred onto scaffold (pink) by making direct contact of the substrate to scaffold. After 18 to 20 h, cells are transferred onto scaffold. **b** Oseoblasts transferred using cell transfer substrate with grid patterning. Bar = 100 μm

**Fig. 2:**
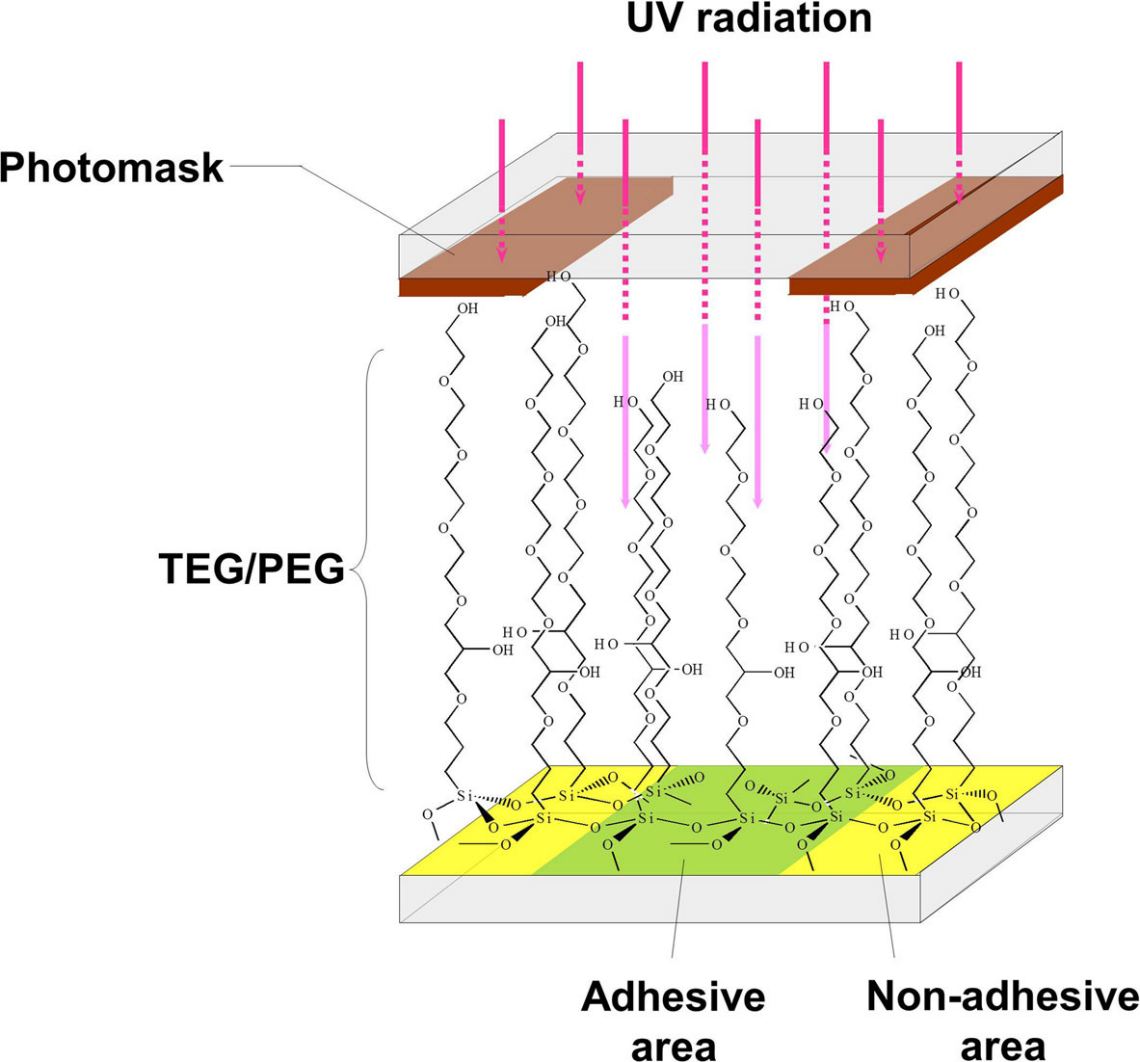
Non-adhesive and adhesive surface on cell transfer substrate. TEG/PEG chains are degraded by UV irradiation. Masked surface with preserved TEG/PEG layer is hydrophobic and cell non- adhesive. Non-masked area, where TEG/PEG is degraded, is hydrophilic and cell adhesive

We have developed “cell transfer technology” that enables transfer of cultured cells onto the surface of transplantation scaffold. Figure 1 shows a schematic diagram of the cell transfer process by cell transfer technology. First, we made thin layer of tetraethyleneglycol (TEG) or polyethyleneglycol (PEG) on glass substrate. Next, we applied photomask on TEG/PEG layer and it was exposed to ultraviolet light. Ultraviolet irradiation partially collapses TEG/PEG chain and made the difference in the length of remaining TEG/PEG chain between photo-masked and non-masked surface. The remaining length of TEG/PEG appears as the difference between hydrophilicity and hydrophobicity of the substrate surface. This difference is involved in the strength of cell adhesion to the substrate surface (Fig. 2). Area with disrupted TEG/PEG is cell adhesive and area with preserved TEG/PEG by photomasking is non-adhesive. Using this difference in hydrophilicity/hydrophilicity, it is possible to stick cells on substrate according to various patterns made by photomasking. Figure 1b demonstrates PKH26-labeled osteoblasts adhered to substrate with grid-like patterning. After adhesion of cells onto substrate, the substrate was placed onto scaffold material making direct contact of the cell surface to scaffold. Eighteen to 24 h later, cells were transferred onto scaffold upon removal of the substrate. The transfer substrate was easily removed from scaffold without any disturbance to the cells. In this step, the strength of substrate-cell adhesion must be less than that between carrier and cells. This can be controlled by the strength and duration of the UV irradiation on TEG/PEG surface after masking. The degradation rate of PEG/TEG can be optimized to maximize the cell transfer efficiency. After removal of the transfer substrate from the scaffold, cells were transferred onto the scaffold surface and were then ready for transplantation.

For surface coating of the transfer substrate, we first coated glass the plate with fluoroalkyl silane (FAS). However, because of the lower stability of patterning on the substrate, FAS was replaced with PEG/TEG. We observed longer stability of patterns made with PEG/TEG on the substrate compared with those made with FAS. The time needed for cell transfer was also found to be shorter for PEG/TEG substrate than that with FAS.

### Amnion as a scaffold

Using cell transfer technology, ex vivo expanded cells were transferred to scaffold materials including hydrogels. Among these, we have used amnion, a part of amniotic membrane, as a scaffold material for cell transfer because of its elasticity, flexibility, and high success rate of cell transfer. Amniotic membrane is the biological membrane forming amniotic sac, which keeps and protects amniotic fluid and embryo inside [4]. Amniotic membrane could be obtained at the time of delivery; however, it is discarded in general. The membrane is composed of amnion and chorion [5]. Amnion is the inner layer of the amniotic membrane. Amnion has been used to treat dermal burns and ulceration, necrosis, and severe inflammation of eyes as dressing material, taking advantage of its anti-microbial and anti-fibrosis property [6, 7]. We isolated amnion from egg membrane by the removal of chorion, and cell components were removed from amnion by high-hydrostatic pressure treatment [8, 9]. The resulting decellurized amnion was used for cell transfer. Considering clinical applications, further evaluations are needed to determine the safety of the membrane in humans.

Almost 100% of cells on transfer substrate were successfully transferred onto amnion after several hours of incubation. Amnion is great scaffold material for cell transfer, as to facilitate high transfer efficiency. Moreover, the most prominent characteristics of amnion upon cell transfer is the stability of transferred cells on the membrane [10, 11]. The transferred cells on amnion are firmly adhered to amnion surface, and this makes it possible to deform and trim the membrane with surgical instruments [10, 11]. This unique characteristic allows easy and reliable cell transplantation.

### Patterned and layered cell transfer

One of the notable features of photolithography is precise transfer of substances according to the fine pattering drawn on substrate. Taking advantage of this unique characteristic of photolithography, we are able to transfer cells in any desired patterns onto scaffold. We made patterns resembling to capillaries on glass substrate and tried to make capillaries by tissue engineering approach. We seeded bovine carotid artery endothelial cells (BCAECs) on transfer substrate with capillary-like pattering and transfer them onto amnion [12]. BCAECs, transferred onto amnion, showed capillary-like structure, and it was also revealed in electron microscopy that the capillary was consisted of vessel wall by BCAECs and lumen inside. We transplanted the BCAEC-transferred amnion into mouse auricle and found that the capillary was kept its structure until 5 days after transplantation. In vivo imaging demonstrated that the capillary functioned in host mouse ear. It is thus conceivable that mimicking the anatomical structure of target tissue, prior to transplantation, may favor the results of cell transplantation. Microvasculature is one good example of this type of cell transplantation.

On the other hand, we can also fabricate cell transfer substrate with whole cell adhesive characteristics. With this substrate, cells are transferred onto amnion in layer structure. In case of cell transplantation requires cell numbers, not positioning, this sheet-like cell transplantation material is useful. We succeeded in transfer of cells and fabricating cell sheet-like materials using various cell types such as fibroblasts, mesenchymal stem cells (MSC), osteoblasts, and endothelial cells [10–13]. Approximately, 5 × 10^5^ cells/cm^2^ were transferred onto an amnion using the cell transfer technology. Furthermore, we transferred two different cell types in overlapping two layers using cell transfer technology and named it “double cell transfer” [11]. For this double cell transfer, we cultured two cell types on transfer substrate and transfer them through single transfer process. This method was applicable to three different cell types and triple cell layers were successfully fabricated. This multiple cell transfer may enable unique cell transplantation considering three-dimensional cell structure and cell-cell communication.

Various cell patterning and cell sheet formation methods have been reported. For cell patterning, two modalities have been mainly studied. One is to utilize the specificity of cell adhesion to the extracellular matrix for cell placement, and this can form sharp edge of patterns [14, 15]. However, it is difficult to form patterns using more than two types of cells using this method. The other method involves the use of “force” to locate cells, including magnetic, electrokinetic, and fluidic forces [16–18]. These methods allow manipulation of large cell numbers, but some of them need labeling of cells and may affect cell viability. The use of temperature responsive polymers has been studied and reported for cell sheet formation [19]. Cell sheets made through this method are too fragile to directly manipulate with surgical instruments. Our method generates a cell sheet by transferring a single cell layer onto a scaffold surface and endows physical strength that achieves stable cell transplantation.

### Cell transplantation using cell transfer technology in animal models

#### Ischemic vascular disease

**Fig. 3:**
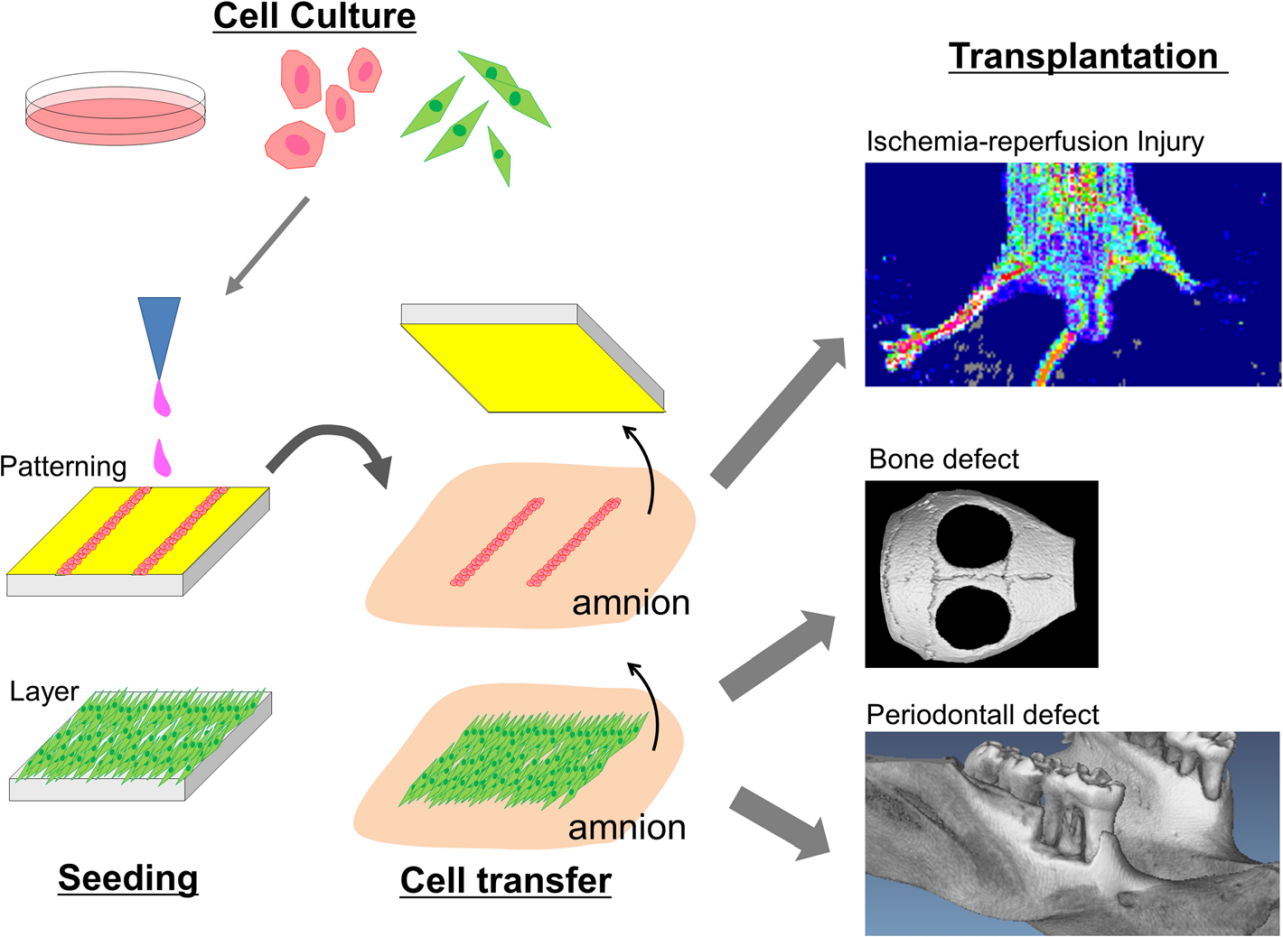
Schema of cell transplantation by cell transfer technology. Cells are transferred onto amnion surface using either patterning or layer cell transfer substrate. Cell-transferred amnion is trimmed and transplanted into animal models such ischemia-reperfusion injury, calvaria bone defect, and periodontal defect model

Ischemic vascular diseases, including myocardial infarction, cerebral infarction, and peripheral arterial embolism, develop by the absence of blood flow due to blockage of the artery and veins [20, 21]. Arterial bypass graft surgery or percutaneous endovascular angioplasty has been performed to restore blood flow for these diseases [22, 23]. Recently, many studies have reported that ischemic conditions were improved by the injection or transplantation of various types of cultured cells including bone marrow-derived mononuclear cells, hematopoietic stem cells, vascular endothelial cells, and endothelial progenitor cells [24, 25]. We fabricated endothelial cell-transferred amnion using transfer substrate with capillary patterning and examined its possibility for the treatment of ischemic diseases by transplanting it into limb ischemia mouse model (Fig. 3) [12]. Reperfusion of ischemic limb was observed after the transplantation of endothelial cell-transferred amnion in laser doppler blood flow and necrosis of the lower limb and gait disturbance were improved. Transplanted capillary fused with the host vascular networks and functioned 5 days after the transplantation. This capillary transplantation is an intermediate method between conventional bypass surgery and cell transplantation and could be new therapeutic option for ischemic disease. To make more stable transplantable capillary, some modifications have been under investigation such as formation of endothelial cell capillary with mural cells.

#### Bone defect

For bone defects caused by tumor resection and fracture, various treatment strategies have been investigated including local application of growth factor with bone-forming activity such as bone morphogenetic proteins and transplantation of autologous or allogeanic bone graft and artificial bone substitute materials such as hydroxyapatite and beta-tricalcium phosphate [26, 27]. Recently, the therapeutic potential of cell transplantation was proposed for bone defect and regeneration of bone defect have been reported by the transplantation of MSC and osteoblasts [28, 29]. We made transfer substrate with entire cell adhesive surface and transferred mouse osteoblasts (Kusa-A1) onto amnion (Fig. 3) [13]. Mouse calvaria bony defect was created, and the osteoblast-transferred amnion was placed to cover the defect. Approximately, 7.8 × 10^4^ cells were transplanted per defect. After 5 weeks, complete closure of bone defect was observed in osteoblast-transplanted defect while control defect did not show the healing. Compared with other test groups including injection of osteoblast, only amnion transplantation, and no treatment, significant bone regeneration was found in osteoblast-transferred amnion defects. Transplanted osteoblasts were found around newly formed bone 5 weeks post operation, and it is suggested that those osteoblasts are directly involved in the regeneration of bone. The firm adhesion of osteoblasts to amnion may contribute to the longer cell retention in bone defects and the prominent bone regeneration observed in osteoblast-amnion transplantation compared with that in cell injection. These results suggested the therapeutic potential of osteoblast-transferred amnion for the treatment of bone defect. We also observed the improvement in bone healing by transplanting double cell-transferred amnion, made using osteoblasts and MSC from periodontal ligament (periodontal ligament stem cells, PDLSC) [11]. We transferred human primary osteoblasts and PDLSC onto amnion to form osteoblast-PDLSC layers on the membrane and transplanted them into mouse calvarial bone defect model. Eight weeks after the transplantation, we observed the enhanced bone regeneration in osteoblast-PDLSC transplanted defects compared with single cell transplanted (osteoblasts or PDLSC alone) defects. These results suggested the clinical feasibility of cell transfer technology in bone regeneration.

#### Periodontal defect

Periodontal disease is characterized by the chronic inflammation and destruction of tooth supporting tissues including bone, periodontal ligament, and cementum mainly due to infection of gram-negative bacteria [30]. Conventional periodontal treatments consisted of mechanical removal of bacterial factors, and it leads to reduction of inflammation and stability of disease status [31]. However, reconstruction of tooth supporting tissues, lost by the disease progression, was hardly observed. Although several regenerative approaches have been applied clinically, sufficient regeneration has not yet been achieved. Recently, it has been demonstrated that cell transplantation is effective in regeneration of periodontal tissues, including bone, periodontal ligament, and cementum, using bone marrow-derived MSC, adipose-derived MSC, PDLSC, and periosteum-derived cells [32]. We made periodontal defect model by removing bone, cementum, and periodontal ligament in rat maxillary molar and transplanted cells using cell transfer technology (Fig. 3) [10]. As a cell type for transplantation, we selected PDLSC because they have been shown to possess the differentiation capacity into various linages of cells such as osteoblast, adipocyte, chondrocyte, and cementoblast [33]. We examined the regenerative potential of PDLSC-transferred amnion (PDLSC-amnion) by transplanting it into surgically created periodontal defect and compared with the defect transplanted with amnion alone. After 4 weeks of healing period, enhanced periodontal tissue regeneration was observed in PDLSC-amnion transplanted defects. Newly regenerated cementum, periodontal ligament, and bone were observed in histological sections. These results suggested that transplantation of PDLSC-amnion could be a novel periodontal regenerative therapy.

## Conclusion

We developed novel cell transplantation method “cell transfer technology” using photolithography technique. By transplanting cell transferred-amnion made by the method, we have demonstrated the therapeutic potential of the material in cell-based treatment including reperfusion of ischemic limb and regeneration of bone and periodontal tissues. Forming a fine patterning by cultured cell is the most unique characteristics of cell transfer technique. In this regard, capillary patterning grafting is taking full advantage of this technology. However, the cell transplantation, which requires cell patterning, might be rather limited. Therefore, cell sheet-like amnion, which we used for bone and periodontal defects, maybe widely applicable for tissue regeneration. Depending on the target tissue to be regenerated, we can select patterned or non-patterned cell transfer substrate. We also found that the transferred cells are firmly and stably adhered on amnion and withstand against the deformation and movement of the membrane by surgical manipulations. Additionally, the flexibility of amnion enables us to transplant cultured cell in direct contact with defects or tissue surface, which is vital importance in certain regenerative cases. Taking these advantages and unique features, cell transfer technology is applicable to wide range of regenerative medicine in the future.

## Abbreviations

BCAEC: Bovine carotid artery endothelial cell
MSC: Mesenchymal stem cells
PDLSC: Periodontal ligament stem cells
PEG: Polyethyleneglycol
TEG: Tetraethyleneglycol

## References

[1] Langer R, Vacanti JP (1993). Tissue engineering. Science.

[2] Howard D, Buttery LD, Shakesheff KM, Roberts SJ (2008). Tissue engineering: strategies, stem cells and scaffolds. J Anat.

[3] Kobayashi A, Miyake H, Hattori H, Kuwana R, Hiruma Y, Nakahama K, Ichinose S, Ota M, Nakamura M, Takeda S, Morita I (2007). In vitro formation of capillary networks using optical lithographic techniques. Biochem Biophys Res Commun.

[4] Niknejad H, Peirovi H, Jorjani M, Ahmadiani A, Ghanavi J, Seifalian AM (2008). Properties of the amniotic membrane for potential use in tissue engineering. Eur Cell Mater.

[5] Toda A, Okabe M, Yoshida T, Nikaido T (2007). The potential of amniotic membrane/amnion-derived cells for regeneration of various tissues. J Pharmacol Sci.

[6] Lo V, Pope E (2009). Amniotic membrane use in dermatology. Int J Dermatol.

[7] Altan-Yaycioglu R, Akova YA, Oto S (2006). Amniotic membrane transplantation for treatment of symblepharon in a patient with recessive dystrophic epidermolysis bullosa. Cornea.

[8] Fujisato T, Minatoya K, Yamazaki S, Meng Y, Niwaya K, Kishida A, Nakatani T, Kitamura S, Mori H, Matsuda H (2005). Preparation and recellularization of tissue engineered bioscaffold for feat valve replacement. Cardiovascular Regeneration Therapies Using Tissue Engineering Approaches.

[9] Wilshaw SP, Kearney JN, Fisher J, Ingham E (2006). Production of an acellular amniotic membrane matrix for use in tissue engineering. Tissue Eng.

[10] Iwasaki K, Komaki M, Yokoyama N, Tanaka Y, Taki A, Honda I, Kimura Y, Takeda M, Akazawa K, Oda S, Izumi Y, Morita I (2014). Tissue Eng Part A..

[11] Akazawa K, Iwasaki K, Nagata M, Yokoyama N, Ayame H, Yamaki K, Tanaka Y, Honda I, Morioka C, Kimura T, Komaki M, Kishida A, Izumi Y, Morita I (2016). Sci Rep.

[12] Akahori T, Kobayashi A, Komaki M, Hattori H, Nakahama K, Ichinose S, Abe M, Takeda S, Morita I (2010). Implantation of capillary structure engineered by optical lithography improves hind limb ischemia in mice. Tissue Eng Part A.

[13] Tsugawa J, Komaki M, Yoshida T, Nakahama K, Amagasa T, Morita I (2011). Cell-printing and transfer technology applications for bone defects in mice. J Tissue Eng Regen Med.

[14] Fukuda J, Khademhosseini A, Yeh J, Eng G, Cheng J, Farokhzad OC, Langer R (2006). Micropatterned cell co-cultures using layer-by-layer deposition of extracellular matrix components. Biomaterials.

[15] Zhang S, Yan L, Altman M, Lässle M, Nugent H, Frankel F, Lauffenburger DA, Whitesides GM, Rich A (1999). Biological surface engineering: a simple system for cell pattern formation. Biomaterials.

[16] Chiu DT, Jeon NL, Huang S, Kane RS, Wargo CJ, Choi IS, Ingber DE, Whitesides GM (2000). Patterned deposition of cells and proteins onto surfaces by using three-dimensional microfluidic systems. Proc Natl Acad Sci U S A.

[17] Ozkan M, Pisanic T, Scheel J, Barlow C, Esener S, Bhatia SN (2003). Electro-optical platform for the manipulation of live cells. Langmuir.

[18] Chiou PY, Ohta AT, Wu MC (2005). Massively parallel manipulation of single cells and microparticles using optical images. Nature.

[19] Elloumi-Hannachi I, Yamato M, Okano T (2010). Cell sheet engineering: a unique nanotechnology for scaffold-free tissue reconstruction with clinical applications in regenerative medicine. J Intern Med.

[20] Schaller B, Graf R (2004). Cerebral ischemia and reperfusion: the pathophysiologic concept as a basis for clinical therapy. J Cereb Blood Flow Metab.

[21] Shimokawa H, Yasuda S (2008). Myocardial ischemia: current concepts and future perspectives. J Cardiol.

[22] Ferket BS, Spronk S, Colkesen EB, Hunink MG (2012). Systematic review of guidelines on peripheral artery disease screening. Am J Med.

[23] Allemang MT, Rajani RR, Nelson PR, Hingorani A, Kashyap VS (2013). Prescribing patterns of antiplatelet agents are highly variable after lower extremity endovascular procedures. Ann Vasc Surg.

[24] Aranguren XL, Verfaillie CM, Luttun A (2009). Emerging hurdles in stem cell therapy for peripheral vascular disease. J Mol Med.

[25] Sieveking DP, Ng MK (2009). Cell therapies for therapeutic angiogenesis: back to the bench. Vasc Med.

[26] Dimitriou R, Jones E, McGonagle D, Giannoudis PV (2011). Bone regeneration: current concepts and future directions. BMC Med.

[27] McAllister BS, Haghighat K (2007). Bone augmentation techniques. J Periodontol.

[28] Colnot C (2009). Skeletal cell fate decisions within periosteum and bone marrow during bone regeneration. J Bone Miner Res.

[29] Akahane M, Nakamura A, Ohgushi H, Shigematsu H, Dohi Y, Takakura Y (2008). Osteogenic matrix sheet-cell transplantation using osteoblastic cell sheet resulted in bone formation without scaffold at an ectopic site. J Tissue Eng Regen Med.

[30] Pihlstrom BL, Michalowicz BS, Johnson NW (2005). Periodontal diseases. Lancet.

[31] Graziani F, Karapetsa D, Alonso B, Herrera D (2017). Nonsurgical and surgical treatment of periodontitis: how many options for one disease?. Periodontol 2000.

[32] Ishikawa I, Iwata T, Washio K, Okano T, Nagasawa T, Iwasaki K, Ando T (2009). Cell sheet engineering and other novel cell-based approaches to periodontal regeneration. Periodontol 2000.

[33] Seo BM, Miura M, Gronthos S, Bartold PM, Batouli S, Brahim J, Young M, Robey PG, Wang CY, Shi S (2004). Investigation of multipotent postnatal stem cells from human periodontal ligament. Lancet.

